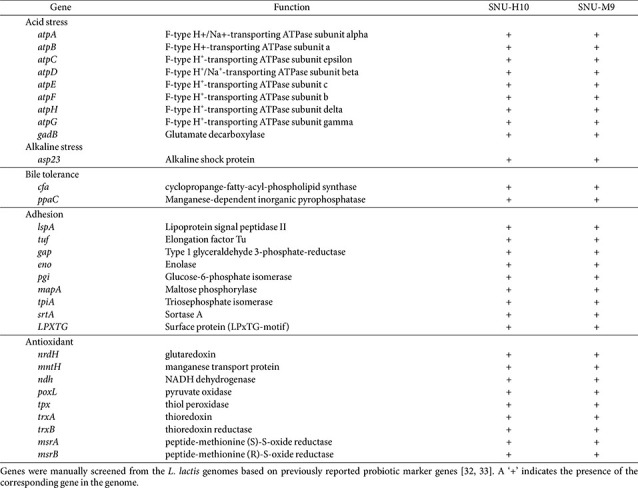# Probiogenomic and Functional Profiling of the Novel Human Gut-Derived Probiotic Strain *Lactococcus lactis* SNU-H10 with Multifunctional Traits

**DOI:** 10.4014/jmb.2601.01069

**Published:** 2026-04-27

**Authors:** Junbum Lee, Jeongheon Choi, Hyokeun Song, Seongbeom Cho

**Affiliations:** 1 College of Veterinary Medicine and Research Institute for Veterinary Science, Seoul National University, Seoul 08826, Republic of Korea; 2 College of Pharmacy, Wonkwang University, Iksan-si, Jeonbuk-do 54538, Republic of Korea

**Keywords:** *Lactococcus lactis*, Probiotics, Whole-genome sequencing, Probiogenomics

## Abstract

*Lactococcus lactis* is a lactic acid-producing bacterium that is globally recognized as safe and is widely used in the food industry, with certain strains exhibiting probiotic properties. Although most studies on *L. lactis* have focused on strains isolated from dairy products, bacteria derived from the gastrointestinal tract may possess traits that enhance their survival and colonization in the gut environment. In this study, we investigated the probiotic potential of human gut-derived *L. lactis* SNU-H10 using probiogenomic and functional profiling. SNU-H10 exhibited tolerance to simulated gastric and intestinal fluids, bile salt exposure, and *in vitro* gut adhesion potential. Safety evaluation confirmed the absence of hemolysis, bile salt hydrolase activity, supported by the lack of genes associated with these traits. SNU-H10 exhibited multifunctional probiotic traits, including *in vitro* free radical scavenging activity, enhanced survival rate of *Caenorhabditis elegans* under oxidative stress, and the ability to synthesize 18 amino acids including branched-chain amino acids (BCAAs). Additionally, probiogenomic analysis revealed that SNU-H10 carried genes related to antioxidant activity (including *nrdH*, *trx*, and *msrA*), the putative production of secondary metabolites (nisin, beta-lactone, and type III polyketide synthase), and the biosynthetic pathways for all three BCAAs. Furthermore, comparative genomic analysis showed that genes related to adhesion (LPxTG-motif protein), nisin biosynthesis, and genes needed for BCAA production were more prevalent in human-derived strains than in those isolated from dairy products. These findings suggest that the human gut-derived *L. lactis* SNU-H10 is a promising probiotic candidate, exhibiting stress tolerance, gut adhesion potential, and multifunctional probiotic traits.

## Introduction

Probiotics are live microorganisms that, when administered in adequate amounts, confer health benefits to the host, particularly within the gastrointestinal (GI) tract [1]. Various mechanisms by which probiotics exert their effects have been studied, including interactions with immune cells, inhibition of pathogenic bacteria, production of beneficial metabolites, support of gut barrier integrity, and regulation of GI hormones and the gut–brain axis [2, 3]. Over the past decades, research on probiotics has advanced significantly. Numerous strains with physiological effects and mechanisms of action have been documented, and some are currently used in commercial applications [4]. However, probiotics often encounter significant challenges for *in vivo* applications. One major obstacle is the colonization resistance offered by the host’s resident gut microbiota, which limits the ability of exogenous probiotics to establish and persist in the GI tract [5]. Therefore, in the development and selection of probiotic strains, exploring microbial sources that are inherently adapted to the GI environment could be a promising strategy because such strains are more likely to successfully colonize the GI tract. Several studies on humans and animals have investigated gut-derived probiotic candidates and reported that these strains exhibit colonization potential and functional properties, suggesting an evolutionary adaptation to the native intestinal niche [6-8]. These findings suggest that the animal gut microbiome could be a valuable reservoir for the discovery of probiotic candidates [9].

*Lactococcus lactis* is a Gram-positive, facultative anaerobic, non-sporulating lactic acid bacterium (LAB) with hundreds of strains having been isolated to date [10]. Strains of *L. lactis* exhibit considerable genetic diversity and have been isolated from diverse ecological niches, with milk, dairy products, and plant-based materials being the most common sources [11]. *L. lactis* is globally recognized as a safe microorganism and has been widely studied for its probiotic applications [10]. However, most studies have focused on strains originating from milk, dairy products, plant materials, and fermented foods [12-16]. In contrast, research on the probiotic properties of *L. lactis* strains isolated from the GI tracts of mammals remains relatively limited, despite their potential relevance for gut-targeted probiotic development.

Based on shotgun metagenomic analysis, *L. lactis* has been reported as one of the most prevalent LABs in the gut microbiota of healthy humans [17]. Furthermore, several metagenomic studies have reported a decreased abundance of *L. lactis* in the microbiome of patients with disease compared with healthy controls. For instance, the gut microbiome of patients with colorectal cancer showed a significant depletion of *L. lactis* relative to that of healthy individuals [18]. Similarly, microbiome analysis of colorectal cancer tissues revealed that the presence of *L. lactis* was significantly higher in normal colorectal tissues than in tumor tissues, and its abundance was positively correlated with the density of natural killer cells [19]. In another gut microbiome study, patients with cerebrovascular disease exhibited a significant decrease in the abundance of *L. lactis* in the gut ecosystem [20]. Collectively, these findings suggest that *L. lactis* plays a significant role in the human gut by modulating host immunity and physiological functions. Therefore, strains of *L. lactis* isolated from the GI tract may have promising potential for the development of novel probiotics.

In this study, we aimed to evaluate novel probiotic candidate isolated from human. To achieve this aim, we investigated the probiotic characteristics of *L. lactis* SNU-H10, isolated from a healthy human, including its tolerance to simulated GI fluids, bile salt tolerance, auto-aggregation, hydrophobicity, and cell adhesion ability. We further assessed the functional profiles, including antioxidant capacity and metabolite production potential. Additionally, whole-genome sequencing (WGS) was conducted to investigate the genomic features of SNU-H10, including probiogenomic analysis for the identification of probiotic marker genes and comparative genomic analysis with publicly available *L. lactis* genomes from different sources. Collectively, this study provides insights into the probiotic characteristics and multifunctional probiotic traits of the human gut-derived SNU-H10 and supports its application as a probiotic candidate.

## Materials and Methods

### Isolation and Culture Media

The *L. lactis* strain was isolated from the feces of a healthy human volunteer who met the following criteria: no history of chronic inflammatory bowel disease or cancer, and no use of antimicrobials for at least two weeks prior to sample collection. Human sample collection was approved by the Institutional Review Board (IRB) of Seoul National University Hospital (approval number: 10-2022-103). Briefly, fecal samples were incubated in De Man–Rogosa–Sharpe (MRS) broth (Thermo Fisher Scientific, USA) at 37°C for 24 h under anaerobic conditions, followed by streaking onto MRS agar and picking single colonies. The isolate was initially identified as *L. lactis* using Matrix-Assisted Laser Desorption/Ionization for Time-of-Flight Mass Spectrometry (MALDI-TOF MS; Bruker, USA), then confirmed by 16S rRNA gene sequencing. SNU-M9, a mouse gut-derived *L. lactis* strain previously isolated and characterized in our laboratory, was included as a comparator strain for evaluation of probiotic characteristics. The strains were stored at -80°C until further use. For evaluation of probiotic properties, frozen stocks were recovered and subcultured at least twice prior to experiments. Cell-free supernatants (CFSs) were prepared by adjusting bacterial cultures to an optical density at 600 nm (OD_600_) of 1.0, incubating the cultures in MRS broth for 24 h under anaerobic conditions, and then centrifuging at 4000 rpm for 10 min. The supernatants were filtered with a 0.22 μm filter to obtain CFSs, which were stored at -80°C until experiments.

### Gut Adhesion Potential of *L. lactis* Strains

**Tolerance to simulated gastric and intestinal fluid.** The tolerance of *L. lactis* strains to simulated gastric and intestinal fluids was assessed according to the method of Mahajan *et al*., with slight modifications [21]. Briefly, bacterial suspensions were adjusted to an OD_600_ of 1.0. One mililiter (ml) of the bacterial suspension was mixed with 9 ml of simulated gastric fluid (NaHCO_3_ 45 mM, NaCl 125 mM, KCl 7 mM, and pepsin 3 g/L), adjusted to pH 2.5. The mixtures were incubated at 37°C for 3 h under anaerobic conditions. Subsequently, 1 ml of the resulting mixture was transferred to 9 ml of simulated intestinal fluid (pancreatin 0.1% and bovine bile 0.3%) adjusted to pH 8.0 and further incubated at 37°C for 3 h under anaerobic conditions. The count of viable bacteria was measured by plating the mixture on MRS agar before treatment and after incubation in simulated gastric and intestinal fluids. The well-characterized probiotic strain *Lacticaseibacillus rhamnosus* GG (LGG) was included as a reference strain to evaluate and compare the tolerance of *L. lactis* strains to simulated gastric and intestinal fluids.

**Tolerance to bile salt.** Bile salt tolerance was evaluated according to the method of Mahajan *et al*. [21]. Bacterial cultures were adjusted to an OD_600_ of 0.1 in MRS broth supplemented with varying concentrations of bovine bile (0%, 0.1%, 0.2%, 0.4%, 0.8%, 1.0%, and 2.0%) (Thermo Fisher Scientific). Bacterial suspensions in bile-supplemented MRS broth were incubated at 37°C for 18 h under anaerobic conditions, and OD_600_ was measured at 0 h, 2 h, 4 h, 6 h, 8 h, 10 h, 12 h, 14 h, 16 h, and 18 h.

**Auto-aggregation ability.** The auto-aggregation ability was assessed according to the method of Xie *et al*. [22]. Bacterial suspensions were adjusted to an OD_600_ of 1.0 and vortexed for 30 s. The suspensions were then incubated under static conditions at 37°C, and OD_600_ was measured after 2, 5, and 24 h. Auto-aggregation (%) was calculated using the following formula: Auto-aggregation (%) = (1−A_t_/A_0_) × 100, where A0 is the initial absorbance, and At is the absorbance at each time point.

**Hydrophobicity.** The hydrophobicity was evaluated according to the method of Zhong *et al*. [23]. Briefly, bacterial suspensions were adjusted to

an OD_600_ of 1.0 and mixed with an equal volume of solvent (chloroform or xylene; Daejung, Republic Korea). After vortexing for 5 min, the mixtures were incubated at 37°C for 30 min to allow phase separation. The aqueous phase was carefully collected, and its OD_600_ was measured. Hydrophobicity (%) was calculated using the following formula: Hydrophobicity (%) = (1 - A_1_/A_0_) × 100, where A0 is the initial absorbance and A_1_ is the absorbance of the aqueous phase after 30 min.

**Cell adhesion assay.** Caco-2 cells were cultured in RPMI 1640 medium (Thermo Fisher Scientific) supplemented with 10% fetal bovine serum (FBS) (Thermo Fisher Scientific) and 1% penicillin-streptomycin-glutamine (100×; Thermo Fisher Scientific) at 37°C in a CO_2_ incubator. For the adhesion assay, cells were seeded into 96-well culture plates at a density of 2.0 × 10^4^ cells per well and incubated until a 100% confluent monolayer was formed. One day prior to the adhesion assay, cells were washed twice with phosphate-buffered saline (PBS) and the culture medium was replaced with RPMI 1640 supplemented with 10% FBS without antimicrobials. *L. lactis* strains were suspended in antimicrobial-free RPMI 1640 to a final concentration of 1.0 × 10^8^ CFU/ml (CFU, colony-forming units) and inoculated into each well. Subsequently, 0.1 ml of the bacterial suspension was mixed with 0.1 ml of antimicrobial-free medium in each well and incubated at 37°C for 2 h in a CO_2_ incubator. To determine the initial CFU, the bacterial suspension was plated immediately on MRS agar without incubation. After incubation, the medium was removed and the wells were washed twice with PBS. The cell monolayer was detached by adding 50 μl of 0.25% trypsin-EDTA (Thermo Fisher Scientific). The resulting cell suspensions were serially diluted in PBS, plated on MRS agar, and incubated at 37°C for 24 h. The adhesion rate was calculated as the percentage of CFU after the adhesion assay relative to the initial CFU. LGG was included as a reference strain to evaluate and compare the adhesion capacity of *L. lactis* strains.

### Safety Analysis of *L. lactis* Strains

**Antimicrobial susceptibility test.** The minimum inhibitory concentration (MIC) was determined to evaluate the antimicrobial susceptibility of *L. lactis* according to the ISO guidelines [24]. Nine antimicrobials were tested: ampicillin, vancomycin, gentamicin, kanamycin, streptomycin, erythromycin, clindamycin, tetracycline, and chloramphenicol (Sigma-Aldrich, USA). Briefly, 100 μl of two-fold serial dilutions of each antimicrobial were prepared in 96-well plates. The *L. lactis* suspension was adjusted to approximately 3 × 10^8^ CFU/ml in MRS broth and then diluted 1,000-fold. Subsequently, 100 μl of this bacterial suspension was added to each well containing the antimicrobial solutions. The plates were incubated at 37°C for 24 h under anaerobic conditions. Experiments were performed in triplicate. The MIC was defined as the lowest concentration of the antimicrobial that showed no visible bacterial growth. Resistance and susceptibility were determined based on the EFSA cut-off values for *L. lactis* [25].

**Hemolysis and bile salt hydrolase activity assay.** Hemolytic activity was assessed by streaking onto tryptic soy agar supplemented with 5% laked horse blood (Thermo Fisher Scientific), followed by incubation at 37°C for 24 h under anaerobic conditions. Bile salt hydrolase (BSH) activity was evaluated by streaking cultures onto MRS agar supplemented with 0.5% (w/v) bovine bile (Thermo Fisher Scientific) and incubating anaerobically at 37°C for 24 h under anaerobic conditions. The appearance of characteristic opaque white colonies was interpreted as a positive result for BSH activity.

### Evaluation of Antioxidant Activity

***In vitro* antioxidant activity.** The antioxidant activities of bacterial CFS were assessed by measuring their scavenging activity against 2,2-diphenyl-1-picrylhydrazyl (DPPH; Glentham Life Sciences, UK) radicals. Briefly, 100 μl of CFS was mixed with 100 μl of 200 μM DPPH solution in methanol and incubated in the dark at room temperature for 30 min. Absorbance was measured at 517 nm using the MultiSkan SkyHigh microplate Spectrophotometer (Thermo Fisher Scientific). Blank samples were prepared by mixing CFS with methanol and processed under the same conditions. Ascorbic acid 10 μg/ml and 50 μg/ml were used as positive controls. All assays were performed in triplicate. DPPH scavenging activity was calculated using the following formula: DPPH scavenging activity (%) = ((A_0_ – A_1_)/A_1_) × 100, where A_0_ represents the absorbance of the control sample containing only DPPH (with the absorbance of methanol subtracted), and A_1_ represents the absorbance of test samples containing bacterial CFS or the positive control (with the absorbance of methanol subtracted).

***In vivo* antioxidant activity.** The antioxidant activity of bacterial CFS was evaluated *in vivo* using a *Caenorhabditis elegans* model. Briefly, a synchronized population of *C. elegans* were obtained using a bleach solution, and then cultured with *Escherichia coli* OP50 as a food source. The nematodes were pre-treated with 10% CFS for two days prior to oxidative stress induction. At the L4 larval stage, worms were exposed to 5-hydroxy-1,4-naphthoquinone (juglone; Sigma-Aldrich) at a final concentration of 16 μM and incubated for 24 h. Subsequently, the numbers of live and dead nematodes were assessed under a microscope. Nematodes were considered alive if they responded to touching with a sterile loop. For each assay, 20 to 30 nematodes were used, and experiments were performed in triplicate.

### *In vitro* Metabolic Profiles of *L. lactis* Strains

Organic acids in bacterial CFS were analyzed using a Dionex Ultimate 3000 HPLC system (Thermo Fisher Scientific) equipped with a refractive index detector (RefractoMax 520; ERC, Japan) and a UV detector set at 210 nm. Separation was performed on an ICSep Coregel 87H3 column (Concise Separations, USA). 0.01 N H_2_SO_4_ was used as the mobile phase, at a flow rate of 0.5 ml/min. The column temperature was maintained at 40°C, and the total run time was 30 min. Quantification was performed using authentic standards of acetic acid, formic acid, lactic acid, citric acid, malic acid, succinic acid, oxalic acid, fumaric acid, and shikimic acid (≥ 98% purity; various suppliers), as well as a volatile organic acid mixture standard (AccuStandard, USA).

Amino acids in bacterial CFS were analyzed using a Dionex Ultimate 3000 HPLC system (Thermo Fisher Scientific) equipped with a fluorescence (FL) detector and a UV detector. Separation was performed on an Inno C18 column (YoungJin biochrome, Republic of Korea). The mobile phase consisted of phase A with 40 mM sodium phosphate buffer, pH 7.0 (Sigma-Aldrich) and phase B with a mixture of water, acetonitrile, and methanol (in a 10:45:45, v/v/v) (Merck). The column temperature was maintained at 40°C, and the sample temperature at 20°C. The flow rate was set at 1.5 ml/min, with a run time of 35 min. The FL detector was set to excitation/emission wavelengths of 340/450 nm for o-phthalaldehyde (OPA) and 266/305 nm for 9-fluorenylmethyl chloroformate (FMOC) reagents (Agilent Technologies, USA). UV detection was conducted at 338 nm. Quantification was performed using a calibration curve prepared from a 17-component amino acid standard mixture (Agilent Technologies), initially dissolved in 0.1 N HCl and serially diluted to final concentrations of 10, 100, 500, and 1000 pmol/μl.

### WGS Analysis

**WGS and assembly.** Genomic DNA from SNU-H10 was extracted using the DNeasy Blood & Tissue Kit (Qiagen, Germany) according to the manufacturer’s instructions. WGS was performed using the Onso sequencing platform (Pacific Biosciences, USA). Briefly, short-read libraries were prepared from the extracted genomic DNA using the Onso Fragmentation Library Prep Kit (Pacific Biosciences). Library fragment size distribution was assessed using a Bioanalyzer 2100 (Agilent Technologies), and DNA concentrations were quantified using the Onso Library Quant Kit (Pacific Biosciences). Based on the average library size and concentration, libraries were diluted to 1 nM for multiplexing. The multiplexed libraries were further diluted to a final concentration of 20 pM, denatured with 0.1 N NaOH, and sequenced on the Onso platform to generate 2 × 150 bp paired-end reads. *De novo* genome assembly was conducted using Unicycler v0.5.1 (with default parameters) [26].

**Probiogenomic and comparative genomic analysis.** The genomes of *L. lactis* strains were annotated using Rapid Annotation using Subsystem Technology (RAST) server [27] and Prokaryotic Genome Annotation System (Prokka) version 1.12 [28]. *In silico* prediction of probiotic properties was carried out using the ProbioMinServer [29]. Additionally, secondary metabolite biosynthetic gene clusters were predicted using antiSMASH v8.0.4 [30]. Functional annotation of KEGG pathways and enzyme commission (EC) numbers was carried out using eggNOG-mapper v2.1.12 against the eggNOG v5.0.2 database [31]. Probiogenomic analysis was conducted by screening the annotated genomes for previously reported probiotic marker genes [32, 33]. Publicly available *L. lactis* genomes were retrieved from the National Center for Biotechnology Information (NCBI) database (accessed December 17, 2025). Only genomes with a complete assembly level were included, whereas atypical genomes, metagenome-assembled genomes, and strains lacking isolation source information were excluded. A total of 71 publicly available *L. lactis* genomes were selected for comparative genomic analysis and regrouped into categories based on host and isolation source information. Among these, 33 strains were isolated from dairy products, 16 from food, 8 from plants, 4 from humans, 3 from water, 3 from fish, 2 from soil, and 2 from insects. Detailed information on the strains included in the comparative genomic analysis is provided in Supplementary Table S1. The distribution of probiotic marker genes and genes encoding enzymes involved in amino acid biosynthesis across *L. lactis* genomes was analyzed using Roary v3.12.0 (with default parameters) [34]. Antimicrobial resistance (AMR) genes were identified using the Resistance Gene Identifier (RGI) software version 6.0.3 against the Comprehensive Antibiotic Resistance Database (CARD) (CARD Variants v4.0.2) and using the ResFinder version 4.6.0 [35, 36]. The probiotic potential risk score (PPRS) was assessed using ProbioMinServer. PPRS values of ≤ 4 were classified as low-risk, >4 and < 6 as medium-risk, and ≥ 6 as high-risk.

### Visualization and Statistical Analysis

Figures were generated using GraphPad Prism version 10.3.0. Bacterial genomes were visualized using Proksee (accessed January 10, 2026) [37]. Statistical analyses of tolerance to gastric and intestinal fluids, auto-aggregation ability, hydrophobicity, cell adhesion rates, antioxidant activity, and amino acid levels were performed using the Student’s *t*-test in GraphPad Prism. To compare the differences in gene prevalence between human-derived and dairy-derived *L. lactis* strains, a one-tailed Fisher’s exact test was performed using the “fisher.test” function in R version 4.3. A *p*-value < 0.05 was considered statistically significant.

## Results

### Isolation of *L. lactis* from Human

A *L. lactis* strain was isolated from the feces of a healthy human. The isolate was first taxonomically characterized using MALDI-TOF mass spectrometry and confirmed by 16S rRNA gene sequencing. The human gut-derived *L. lactis* strain was designated as SNU-H10, and the probiotic characteristics, antioxidant activity, and *in vitro* metabolic profiles of SNU-H10 were subsequently evaluated.

### Gut adhesion Potential of *L. lactis* Strains

In this study, simulated gastric and intestinal fluids were used to assess the ability of SNU-H10 to tolerate the physiological obstacles encountered by probiotics in the host GI tract (Table 1). After exposure to simulated gastric and intestinal fluids, the log reductions in viable bacterial counts were 2.04 ± 0.31 for SNU-H10 and 2.66 ± 0.10 for SNU-M9, compared with 5.24 ± 0.33 for the reference probiotic strain LGG (Table 1). The log reduction of SNU-H10 was significantly lower than that of LGG (*p* = 0.0003). These results suggest that the gut-derived *L. lactis* strain SNU-H10 can withstand simulated GI conditions, supporting its potential to survive oral administration and reach the intestine.

**Fig. 1:**
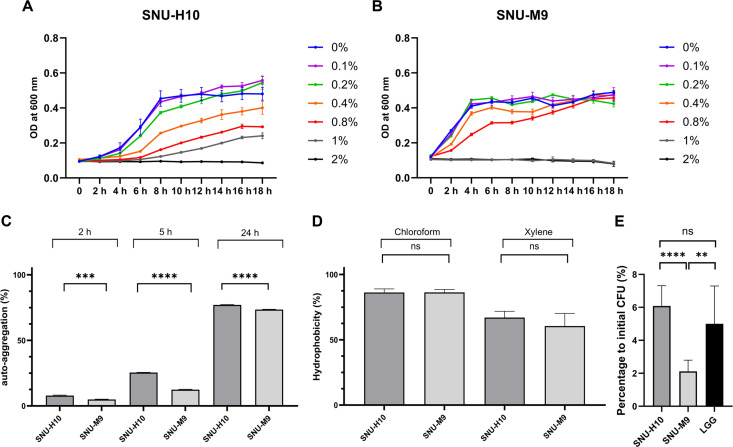
Bile salt tolerance and adhesion properties of SNU-H10. (**A**) Growth curves of *Lactococcus lactis* SNU-H10 in De Man–Rogosa–Sharpe broth supplemented with varying bile concentrations, measured by optical density at 600 nm (OD_600_). Data are presented as mean ± standard deviation (n = 3). (**B**) Growth curve of *L. lactis* SNU-M9 in De Man–Rogosa–Sharpe broth supplemented with varying bile salt concentrations, measured at OD_600_. Data are presented as mean ± standard deviation (n = 3). (**C**) Auto-aggregation ability of SNU-H10 and SNU-M9 measured at 2, 5, and 24 h. Data are presented as mean ± standard deviation (n = 3). (**D**) Hydrophobicity values of SNU-H10 and SNU-M9 using chloroform and xylene as solvents. Data are presented as mean ± standard deviation (n = 3). (**E**) Adhesion rates of SNU-H10, SNU-M9, and LGG to Caco-2 cells. The bar plot represents the proportion of viable bacteria remaining after the adhesion assay compared with the initial viable count (n = 10). Statistical significance: ‘**’ represents significant differences at *p* < 0.01, ‘***’ represents significant differences at *p* < 0.001, and ‘****’ represents significant differences at *p* < 0.0001.

The bile salt tolerance of SNU-H10 was assessed by monitoring its growth in MRS broth supplemented with varying concentrations of bile over an 18 h incubation period. SNU-H10 grew at bile concentrations ranging from 0.1% to 1.0% (Fig. 1A), whereas SNU-M9 grew at bile concentrations ranging from 0.1% to 0.8% (Fig. 1B). The human gut-derived strain SNU-H10 exhibited growth at higher bile salt concentrations than the mouse gut-derived strain SNU-M9.

To evaluate the gut adhesion potential of *L. lactis* strains in the GI tract, auto-aggregation ability, hydrophobicity, and adhesion to Caco-2 cells were assessed. SNU-H10 exhibited auto-aggregation capacities of 7.87% ± 0.004, 25.47% ± 0.001, and 77.01% ± 0.003 at 2, 5, and 24 h, respectively, whereas *L. lactis* SNU-M9 exhibited corresponding values of 4.95% ± 0.002, 12.38% ± 0.003, and 73.49% ± 0.002, respectively (Fig. 1C). The average hydrophobicity obtained using chloroform was 86.19% ± 0.03 for SNU-H10 and 86.27% ± 0.02 for SNU-M9 (Fig. 1D). With xylene, SNU-H10 and SNU-M9 exhibited hydrophobicity values of 67.02% ± 0.05 and 60.48% ± 0.10, respectively (Fig. 1D). The auto-aggregation capacity of the human gut-derived SNU-H10 was significantly higher than that of the mouse gut-derived SNU-M9 at 2, 5, and 24 h (*p* = 0.0004, *p* < 0.0001, and *p* < 0.0001, respectively). The mean adhesion rates to Caco-2 cells were 6.08% ± 1.23% for SNU-H10, 2.11% ± 0.68% for SNU-M9, and 5.00% ± 2.29% for the reference probiotic strain LGG (Fig. 1e). Although the mean adhesion rate of SNU-H10 was numerically higher than that of LGG, the difference was not statistically significant (*p* = 0.2683). Conversely, SNU-H10 exhibited a significantly higher mean adhesion rate to Caco-2 cells compared to SNU-M9 (*p* < 0.0001).

### Safety Analysis

**Fig. 2:**
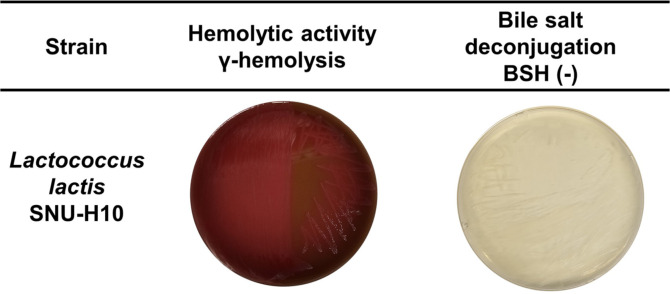
Hemolysis and bile salt hydrolase (BSH) activity of SNU-H10. γ-hemolysis indicates the absence of both α- and β-hemolytic activity. BSH (-) indicates that no characteristic opaque white colonies were observed.

To assess the safety of SNU-H10, its hemolytic activity, BSH activity, and antimicrobial resistance patterns were evaluated. Neither α-hemolysis nor β-hemolysis was observed in either *L. lactis* strain (Fig. 2). When cultured on MRS agar supplemented with bile salts, neither SNU-H10 nor SNU-M9 formed the characteristic opaque, white colonies typically associated with BSH activity, indicating the absence of BSH activity in either strain (Fig. 2).

The antimicrobial susceptibility profiles of *L. lactis* strains, determined using MIC values, showed that both strains were susceptible to seven antimicrobials: ampicillin, vancomycin, gentamicin, kanamycin, erythromycin, clindamycin, and tetracycline. In contrast, both *L. lactis* strains exhibited higher MIC values for streptomycin and chloramphenicol than the cut-off values defined by the EFSA, indicating potential resistance to these antimicrobials (Table 2).

### Antioxidant Activity of SNU-H10

**Fig. 3:**
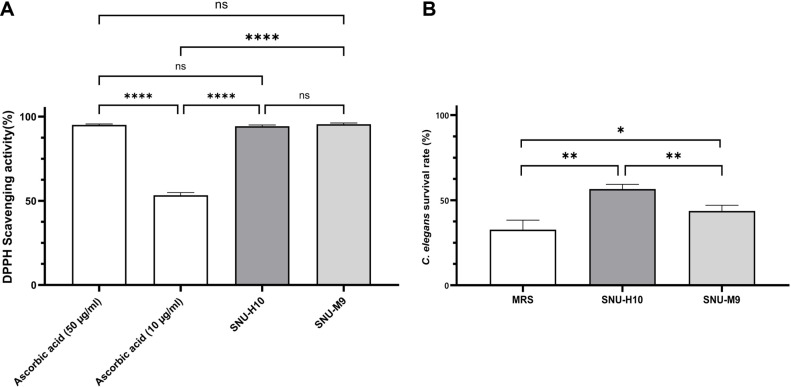
Antioxidant activity of SNU-H10 cell-free supernatants *in vitro* and *in vivo*. (**A**) *In vitro* antioxidant activity measured using 2,2-diphenyl-1-picrylhydrazyl (DPPH) scavenging assay. White bars indicate the scavenging activity of ascorbic acid used as a positive control at concentrations of 10 and 50 μg/mL. Data are presented as mean ± standard deviation (n = 4). (**B**) *In vivo* antioxidant activity assessed by evaluating the survival rates of *Caenorhabditis elegans* exposed to oxidative stress induced by 5-hydroxy-1,4-naphthoquinone (juglone). Data are presented as mean ± standard deviation (n = 3). Statistical significance: ‘*’ represents significant differences at *p* < 0.05, and ‘****’ represents significant differences at *p* < 0.0001.

To evaluate the functional profiles of SNU-H10, the antioxidant activities of its CFS was assessed both *in vitro* using a free radical scavenging assay and *in vivo* using a *C. elegans* oxidative stress model. The DPPH radical scavenging assay revealed that the CFS from both SNU-H10 and SNU-M9 exhibited considerable *in vitro* antioxidant activity (above 90%) (Fig. 3A). The average scavenging rates of the CFS of SNU-H10 and SNU-M9 were 95.47% and 94.28%, respectively. These antioxidant values were significantly higher than the activity of the 10 μg/ml ascorbic acid standard (average scavenging rate, 53.33%) (*p* < 0.0001). When compared with the scavenging activity of 50 μg/ml ascorbic acid (average scavenging rate, 95.13%), the antioxidant activities of SNU-H10 and SNU-M9 CFS were not significantly different (*p* = 0.9579 and *p* = 0.6060, respectively).

To validate the antioxidant effects *in vivo*, oxidative stress was induced in *C. elegans* using juglone, and survival rates were evaluated (Fig. 3B). The average survival rates of *C. elegans* treated with MRS, SNU-H10, and SNU-M9 were 32.73% ± 0.06, 56.59% ± 0.03, and 43.71% ± 0.03, respectively. The CFS of both SNU-H10 and SNU-M9 significantly increased the survival of *C. elegans* under oxidative stress compared with the MRS control (*p* = 0.0026 and *p* = 0.0419, respectively). Notably, the CFS of SNU-H10 significantly enhanced the survival rate of *C. elegans* compared with the CFS of SNU-M9 (*p* = 0.0064).

### *In vitro* Metabolic Profiles of SNU-H10

In the current study, targeted metabolomics was used to identify organic acids and amino acids in the CFSs of SNU-H10 to investigate functional profiles related to metabolite production (Table 3). After 24 h of anaerobic culture, the CFSs of SNU-H10 and SNU-M9 showed significantly higher concentrations of 18 amino acids (aspartic acid, serine, glutamine, histidine, glycine, threonine, citrulline, alanine, γ-aminobutyric acid (GABA), tyrosine, valine, methionine, tryptophan, phenylalanine, isoleucine, ornithine, leucine, and proline) compared with the MRS broth control. Conversely, the concentration of three amino acids, glutamic acid, asparagine, and arginine, significantly decreased. The levels of taurine and lysine in the bacterial CFS were consistent with those in the MRS broth. Among the organic acids, only acetic acid was detected in the CFS of SNU-H10 and SNU-M9 at levels similar to those in the initial MRS broth, suggesting that these *L. lactis* strains did not produce organic acids *in vitro*.

### Genomic Properties of SNU-H10

Among the two *L. lactis* strains evaluated for probiotic characteristics and multifunctional probiotic traits in this study, SNU-H10 tolerated higher concentrations of bile salts, exhibited significantly stronger auto-aggregation, and demonstrated significantly higher antioxidant activity in the *C. elegans* model than SNU-M9. These results suggest that SNU-H10 may have greater survival and gut adhesion potential in the human GI tract and may also confer stronger antioxidant effects *in vivo*. Therefore, WGS was performed for SNU-H10, and probiogenomic analyses were conducted to further explore its potential application as a probiotic.

**Fig. 4:**
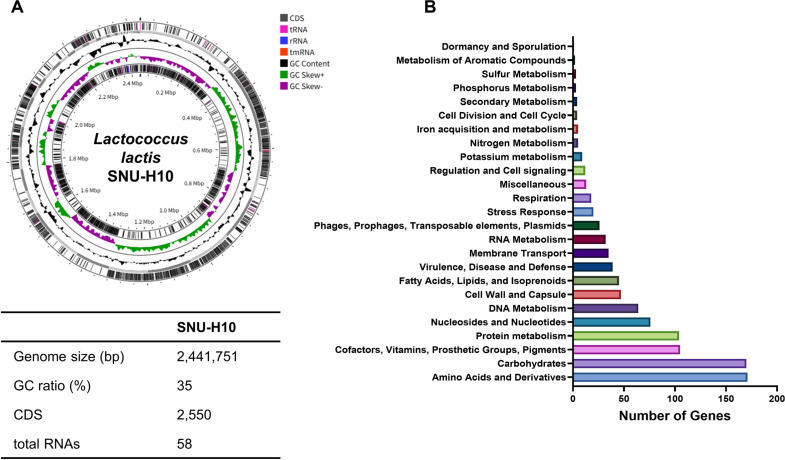
Genomic properties of SNU-H10. (**A**) Circular genome map of *Lactococcus lactis* SNU-H10 visualized using Proksee. From the outer to the inner ring are the positions of protein-coding sequences (CDSs) and RNAs on the forward and reverse strands, GC content, and GC Skew [(G-C)/(G+C)]. Positive GC Skew is shown in green, while negative GC Skew is shown in purple. The table below presents the genomic properties of SNU-H10 identified using the RAST server. (**B**) Bar plot showing the distribution of subsystem categories identified in the genome of SNU-H10 using the RAST server.

RAST annotation revealed that SNU-H10 was 2,441,751 base pairs (bp) in size, with a guanine-to-cytosine (GC) ratio of 35% (Fig. 4A). In SNU-H10, genome annotation predicted 2,550 protein-coding sequences (CDSs) and 58 total RNAs. Genome annotation using the RAST server identified 238 subsystems in SNU-H10 (Fig. 4B). Among these, the subsystem with the highest feature count was related to amino acids and derivatives (n = 171), followed by carbohydrates (n = 170), cofactors, vitamins, prosthetic groups, and pigments (n = 105), and protein metabolism (n = 104). In the SNU-H10 genome, subsystems related to amino acid and protein metabolism were among the most predominant, suggesting that SNU-H10 harbors substantial genetic and metabolic potential for amino acid biosynthesis and utilization.

### Probiogenomic Analysis for Probiotic Marker Genes

Genome-guided analysis was conducted to identify probiotic marker genes associated with the probiotic characteristics of SNU-H10, including stress tolerance, gut adhesion potential, and safety (Table 4). Nine genes previously reported to be involved in acid stress resistance in LAB were identified in the genome of SNU-H10. Among these, eight genes (*atpA*, *atpB*, *atpC*, *atpD*, *atpE*, *atpF*, *atpG*, and *atpH*) encode subunits of the F_0_F_1_-ATP synthase complex, and one gene (*gadB*) encodes glutamate decarboxylase. Additionally, the alkaline shock protein gene *asp23*, which has been associated with tolerance to high pH, was detected in the genome of SNU-H10. Two genes associated with bile salt tolerance were also identified in SNU-H10: *cfa* (cyclopropane-fatty-acyl-phospholipid synthase) and *ppaC* (inorganic pyrophosphatase). Eight adhesion-related genes, including *mapA* (maltose phosphorylase), *lspA* (lipoprotein signal peptidase II), *tuf* (elongation factor Tu), *gap* (type 1 glyceraldehyde 3-phosphate-reductase), *eno* (enolase), *pgi* (glucose-6-phosphate isomerase), *tpiA* (triosephosphate isomerase), and *srtA* (sortase A), were also found in the SNU-H10 genome.

The safety of SNU-H10 as a potential probiotic was predicted using probiogenomic analyses. The PPRS was 4.58, indicating that SNU-H10 poses a medium risk as a probiotic candidate. However, no genes encoding hemolysin or BSH (EC 3.5.1.24) were detected in the SNU-H10 genome, which was consistent with the phenotypic results showing no hemolytic or BSH activity. Regarding AMR genes, only the *lmrD* gene was identified in the SNU-H10 genome using the CARD database, whereas no AMR genes were detected using ResFinder.

**Fig. 5:**
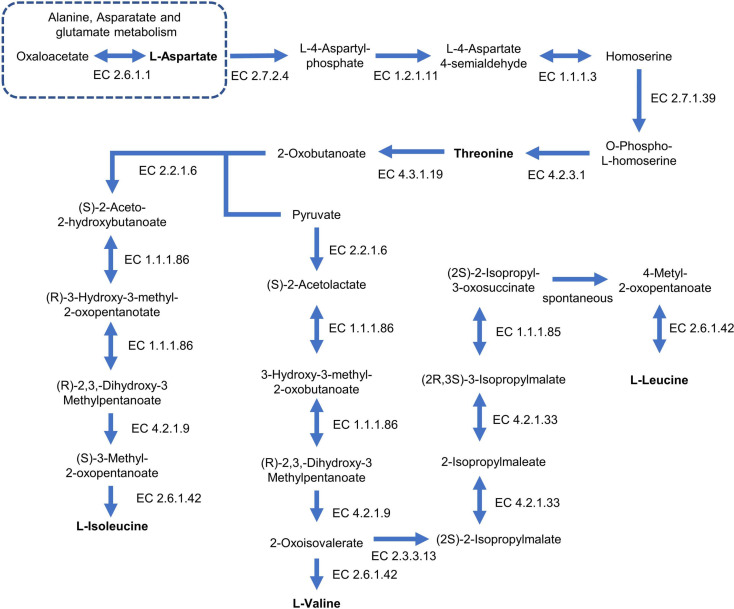
Reconstructed functional pathways for branched-chain amino acid biosynthesis in SNU-H10. Blue arrows and the corresponding Enzyme Commission (EC) numbers indicate enzymes for which the encoding genes were identified in SNU-H10. Metabolites that are amino acids are shown in bold.

To investigate genes related to the multifunctional probiotic traits of SNU-H10, genome mining was conducted to identify genes associated with antioxidant activity, secondary metabolite production, and amino acid biosynthesis. Nine genes related to antioxidant activity were detected in the SNU-H10 genome, including genes encoding thioredoxin system components (*tpx*, *trxA*, and *trxB*), glutaredoxin (*nrdH*), manganese transport protein (*mntH*), NADH antioxidant system (*ndh*), pyruvate oxidase (*poxL*), and methionine sulfoxide reductases (*msrA*, *msrB*). Collectively, these genes may support the *in vitro* and *in vivo* antioxidant activity observed in the current study. SNU-H10 harbored gene clusters associated with several secondary metabolites, including those related to beta-lactone, nisin (lanthipeptide class I), terpene precursors, ribosomally synthesized and post-translationally modified peptides (RiPP), radical S-adenosylmethionine-RiPP (RaS-RiPP), and type III polyketide synthase (T3PKS). The presence of these gene clusters indicates the predicted genetic potential of SNU-H10 to produce secondary metabolites. Genome analysis revealed that SNU-H10 possessed genes encoding all the key enzymes required for the biosynthesis of BCAA from threonine (EC 4.3.1.19, 2.2.1.6, 1.1.1.86, 4.2.1.9, 2.3.3.13, 4.2.1.33, 1.1.1.85, and 2.6.1.42) (Fig. 5). Additionally, the genome of SNU-H10 harbored genes encoding enzymes required for threonine biosynthesis from L-aspartate (EC 2.7.2.4, 1.2.1.11, 1.1.1.3, 2.7.1.39, and 4.2.3.1), as well as genes for the conversion of oxaloacetate from the citrate cycle to L-aspartate via aspartate transaminase (EC 2.6.1.1). These results suggest that SNU-H10 may have a prototrophic potential for BCAA biosynthesis.

### Comparative Genomic Analysis between *L. lactis* Genomes

**Fig. 6:**
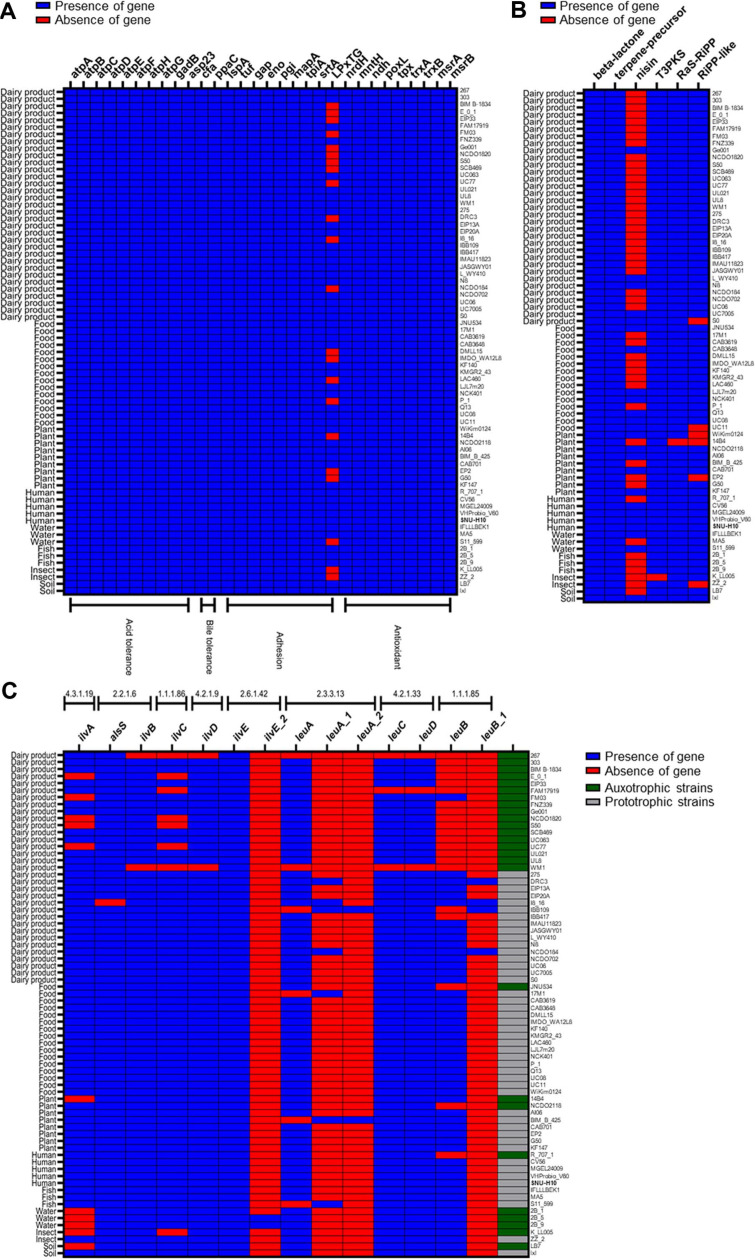
Comparative genomic analysis for the presence and absence of probiotic marker genes and branched-chain amino acid (BCAA) biosynthesis genes among SNU-H10 and 71 *Lactococcus lactis* genomes. (**A**) Heatmap showing the distribution of probiotic marker genes associated with acid tolerance, bile tolerance, adhesion, and antioxidant activity across *L. lactis* genomes. The left panel indicates the isolation source of each strain, the top panel lists the presence or absence of specific genes, and the bottom panel categorizes the genes by their associated probiotic traits. The right panel lists the strain names of *L. lactis* used for comparative genomic analysis. SNU-H10 strain is highlighted in bold. Presence and absence of genes are indicated in blue and red, respectively. (**B**) Heatmap showing the distribution gene clusters related to secondary metabolite biosynthesis across the *L. lactis* genomes. The left panel shows the isolation source of each strain, and the top panel lists the identified secondary metabolites. The right panel lists the strain names of *L. lactis* used for comparative genomic analysis. SNU-H10 strain is highlighted in bold. Presence and absence of genes are indicated in blue and red, respectively. (**C**) Heatmap showing the distribution of genes encoding enzymes involved in BCAA biosynthesis. The left panel indicates the isolation source of each strain, and the top panel shows the gene names and their corresponding enzymes. The right panel lists the strain names of *L. lactis* used for comparative genomic analysis. Strains isolated in this study are highlighted in bold. Presence and absence of genes are indicated in blue and re

A total of 71 complete *L. lactis* genomes were obtained from the NCBI database for comparative genomic analysis with SNU-H10. The distribution of probiotic marker genes, secondary metabolite gene clusters, and amino acid biosynthesis-related genes identified in SNU-H10 was examined among the publicly available *L. lactis* genomes (Fig. 6). All probiotic marker genes associated with pH tolerance, bile salt tolerance, and antioxidant activity that were identified in SNU-H10 were also found in all 71 *L. lactis* genomes analyzed (Fig. 6A). However, one of the gut adhesion potential marker genes, which encodes an LPxTG-motif protein was absent in a subset of *L. lactis* genomes. Specifically, the LPxTG-motif protein gene was not detected in 22 of the 71 strains. These included 12 strains isolated from dairy products (36.36%), four from food products (25%), three from plants (37.5%), two from insects (100%), and one from fish (33.33%). Although the proportion of strains carrying the LPxTG-motif protein gene appeared higher in human-derived strains than in dairy-derived strains, this difference did not reach statistical significance (*p* = 0.1311).

The distribution of secondary metabolite gene clusters identified in the genome of SNU-H10 was examined among 71 *L. lactis* genomes (Fig. 6B). Gene clusters encoding beta-lactone biosynthesis and terpene precursors were consistently present in all 71 *L. lactis* genomes analyzed. In contrast, the T3PKS gene cluster was missing in one strain isolated from an insect. The nisin-biosynthetic gene cluster was absent in 48 of the 71 *L. lactis* genomes. Specifically, the nisin-negative strains included 28 strains from dairy products (84.85%), eight from food products (50%), four from plants (50%), three from water (100%), two from insects (100%), and one strain each from soil (50%), fish (33.33%), and humans (25%). Notably, the proportion of strains possessing the nisin-biosynthetic gene cluster was significantly higher in human-derived strains than in dairy-derived strains (*p* = 0.0075).

Comparative analysis of genes encoding enzymes required for BCAA biosynthesis across the 71 *L. lactis* genomes revealed that 26 strains lacked at least one key enzyme in the metabolic pathway, suggesting potential BCAA auxotrophy (Fig. 6C). Among these 26 strains, 17 were isolated from dairy products (51.52%), followed by three strains from water (100%), two from plants (25%), and one strain each from humans (25%), food (6.25%), soil (50%), and insects (50%). Similar to the observations for the LPxTG-motif protein gene, a higher proportion of putative BCAA-prototrophic strains was observed in human-derived strains compared to dairy-derived strains. However, this trend did not reach statistical significance (*p* = 0.2046).

## Discussion

This study suggests that the human gut-derived SNU-H10 is a promising probiotic candidate with multifunctional traits, including antioxidant activity, the capacity for amino acid biosynthesis, and the potential for secondary metabolite production. As probiotics are typically administered orally, it is essential for them to survive and maintain viability while passing through the GI tract, where they are exposed to physiologically relevant stresses. These include exposure to acidic conditions, digestive enzymes such as pepsin and pancreatin, and bile salts [38-40]. In this study, SNU-H10 demonstrated the ability to survive in simulated gastric and intestinal fluids, and exhibited greater tolerance than the reference probiotic strain LGG. Additionally, SNU-H10 maintained growth at bile salt concentrations higher than 0.3%, which is considered the average physiological bile concentration in the human GI tract [41].

The ability of probiotics to colonize the gut after surviving stressful conditions in the GI tract is considered an important probiotic characteristic. Gut colonization is closely associated with the adhesion capacity of bacteria, which is commonly evaluated *in vitro* by measuring auto-aggregation, hydrophobicity, and adhesion to intestinal epithelial cells [42, 43]. Previous studies have reported that *L. lactis* strains isolated from dairy products and fermented foods exhibit auto-aggregation levels ranging from 15% to 66% [44, 45]. *L. lactis* strains from sources such as crop rice and Swiss cheese showed hydrophobicity in chloroform ranging from 14.9% to 31.72% and in xylene from 25.5% to 78.8% [45-47]. Although there are no established standard values for auto-aggregation and hydrophobicity, high values are generally considered favorable characteristics associated with probiotic potential [48]. In our study, SNU-H10 exhibited auto-aggregation and hydrophobicity values within the upper range reported for *L. lactis* strains, and it also demonstrated an adhesion capacity to Caco-2 cells comparable to that of LGG. Collectively, these results suggest that the human gut-derived strain SNU-H10 may possess strong gut adhesion potential.

The European Food Safety Authority strongly recommends evaluating the hemolytic activity of probiotic candidates [49]. In addition, assessment of BSH activity is also important, as BSH can enhance bile salt tolerance and promote gut colonization, but may also impair lipid digestion and contribute to the formation of potentially toxic secondary bile acids by other gut microbes [50]. In this study, neither hemolytic activity nor BSH activity was detected in SNU-H10, and no genes associated with these traits were identified, supporting the safety of this *L. lactis* strain. Regarding AMR, SNU-H10 carried the ATP-binding cassette multidrug transporter gene *lmrD*. This multidrug efflux pump gene may be associated with the observed MIC patterns, such as the elevated MIC values for streptomycin and chloramphenicol that exceeded the EFSA cut-off values established for *L. lactis*. However, as *lmrD* is chromosomally encoded and is considered an intrinsic resistance determinant in *L. lactis*, it poses a low risk of horizontal transfer via plasmids [51]. Additionally, this gene has been implicated in bile salt resistance mechanisms in *L. lactis* [52], which may contribute to the bile salt-tolerant phenotypes observed in SNU-H10.

With the increasing availability of whole-genome data for diverse probiotic strains, probiogenomics has emerged as a valuable approach that uses the genetic blueprints to predict and evaluate probiotic potential [53]. Probiogenomic analysis revealed that SNU-H10 harbors multiple probiotic marker genes that may be linked to its multifunctional probiotic traits observed *in vitro* and *in vivo*, particularly antioxidant activity and amino acid biosynthesis. Specifically, SNU-H10 harbored marker genes implicated in oxidative stress defense, which could support the antioxidant effects observed in this study. In addition, genes encoding enzymes involved in amino acid biosynthesis, including BCAAs, were identified, supporting the amino acid production profiles detected in this study. Moreover, SNU-H10 carried biosynthetic gene clusters associated with secondary metabolites, suggesting the potential to produce lanthipeptide class I, beta-lactone, terpene precursors, and T3PKS-derived products. The class I lanthipeptide in *L. lactis* is also known as nisin, a bacteriocin produced by *L. lactis* that is widely used as a food preservative because of its ability to inhibit pathogenic bacteria [54]. Another potential secondary metabolite, beta-lactone, functions as an antimicrobial agent by inhibiting homoserine transacetylase and disrupting the methionine biosynthesis pathway in other bacteria [55]. Terpenes have attracted attention owing to their therapeutic potential against AMR bacteria by disrupting membrane integrity through interactions with polysaccharides, fatty acids, phospholipids, and proteins [56]. T3PKS-derived products have been reported to serve as precursors for UV-protective pigments and antimicrobials, and facilitate respiration under hypoxic conditions [57]. The presence of these gene clusters in the SNU-H10 genome highlights its predicted genetic potential for secondary metabolite production. Comparative genomic analysis of SNU-H10 and 71 publicly available *L. lactis* genomes revealed that most probiotic marker genes were present across all the analyzed strains. However, the gene encoding the LPxTG-motif protein exhibited a varying distribution pattern. The LPxTG-motif proteins are cell wall-anchored surface proteins found in Gram-positive bacteria and have been reported to enhance bacterial adhesion to epithelial cells [58]. LAB strains expressing LPxTG have been reported to exhibit improved colonization and prolonged residence in the host GI tract [58]. The presence of the LPxTG-motif protein gene in SNU-H10 suggests strong adhesion potential, which may contribute to effective colonization of the human GI tract. Although the proportion of strains carrying the LPxTG-motif protein gene was higher in human-derived *L. lactis* strains (100%) compared to dairy-derived strains (63.64%), this difference was not statistically significant. Nevertheless, the presence of this gene in all analyzed human-derived strains, including SNU-H10, suggests a potentially favorable adhesion profile that might support colonization in the human GI tract, although further validation with a larger number of human-derived strains may be needed.

In this study, our findings suggest that SNU-H10 exhibits putative prototrophy for BCAA biosynthesis. BCAAs (valine, leucine, and isoleucine) are essential amino acids that cannot be synthesized endogenously in humans. Nevertheless, they play critical roles as building blocks for protein synthesis, promote glycogen uptake and synthesis, and support the proper function of immune cells [59-61]. In addition to these physiological roles, BCAAs also serve as important precursors of volatile compounds that contribute to the flavor of dairy products [62]. Our comparative genomic analysis revealed that more than half (51.52%) of the *L. lactis* strains originating from dairy products were genetically predicted to be auxotrophic for BCAA biosynthesis, indicating the absence of at least one essential gene within the metabolic pathways for the biosynthesis of all three BCAAs. While the proportion of putative BCAA prototrophy appeared higher in human-derived strains than in dairy-derived strains, this trend did not reach statistical significance. Nevertheless, these observations are in line with those of a previous report indicating that *L. lactis* strains of dairy origin are often putatively auxotrophic for BCAA biosynthesis, whereas strains from other sources tend to be putatively prototrophic [63]. Similarly, the nisin biosynthetic gene cluster was absent in a larger proportion of *L. lactis* strains isolated from dairy products (84.85%) than in human-derived strains (25%). Together, these findings suggest that human gut-derived *L. lactis* strains may possess beneficial functional traits, such as potential nisin production, which are not consistently present in dairy-derived *L. lactis*. This highlights the need to expand the search for probiotic candidates beyond traditional dairy sources.

The *C. elegans* model is considered a valuable *in vivo* system for evaluating antioxidant effects, as it shares highly conserved antioxidant and anti-aging pathways with humans [64]. Furthermore, *C. elegans* has an estimated 60–80% of genes with homologues in humans, highlighting its value as a model for identifying potential therapeutic targets [65]. However, the *C. elegans* model has a relatively simple anatomy and lacks the complex organs and tissues found in mammals [66]. Consequently, the *in vivo* antioxidant properties of SNU-H10 observed in this nematode model may not directly translate to human health. Therefore, further investigations using mammalian models may be needed to validate the potential health benefits of this probiotic candidate and its application in humans. In conclusion, this study investigated the probiotic properties of the human gut-derived *L. lactis* SNU-H10. Our findings showed that SNU-H10 possesses major probiotic characteristics, including acid and bile salt tolerance, gut adhesion potential, and safety. In addition, SNU-H10 exhibited multifunctional probiotic traits, such as antioxidant activity and the capacity for amino acid biosynthesis, including BCAAs. Probiogenomic analyses further suggested the potential for secondary metabolite production and putative prototrophy for BCAA biosynthesis. Furthermore, comparative genomic analysis revealed that the proportion of *L. lactis* strains carrying the nisin-biosynthetic gene cluster was higher among human-derived strains than among dairy-derived strains. Collectively, these characteristics support the potential of SNU-H10 as a promising probiotic candidate.

## Supplemental Materials

Supplementary data for this paper are available on-line only at http://jmb.or.kr.



## Figures and Tables

**Table 1:** Survival assay of SNU-H10 under exposure to simulated gastric and intestinal conditions.

	Initial colony count (log CFU/mL)	Colony count after incubation (log CFU/mL)	Log reduction
SNU-H10	8.92 ± 0.35	6.88 ± 0.10	2.04 ± 0.31
SNU-M9	8.28 ± 0.15	5.62 ± 0.17	2.66 ± 0.10
LGG^a^	10.52 ± 0.18	5.28 ± 0.15	5.24 ± 0.33

Colony counts were analyzed before and after incubation in simulated gastric and intestinal fluids, and the colony-forming unit (CFU) values were log-transformed. Data are presented as mean ± standard deviation (n = 3). Log reduction was calculated by subtracting the colony count after incubation (log CFU/mL) from the initial colony count (log CFU/mL).

^a^LGG: *Lacticaseibacillus rhamnosus* GG

**Table 2:** Antimicrobial susceptibility of SNU-H10 determined by minimum inhibitory concentration values.

Antimicrobials	Ampicillin	Vancomycin	Gentamicin	Kanamycin	Streptomycin	Erythromycin	Clindamycin	Tetracycline	Chloramphenicol
EFSA cut-off values^a^	2	4	32	64	32	1	1	4	8
SNU-H10	0.5	2	26.7	42.7	149.3	0.5	0.5	0.5	13.3
Resistance^b^	S	S	S	S	R	S	S	S	R
SNU-M9	0.5	0.8	16	26.7	84	0.5	0.5	0.5	13.3
Resistance^b^	S	S	S	S	R	S	S	S	R

^a^The minimum inhibitory concentration (MIC) values were determined in accordance with the European Food Safety Authority (EFSA) guidelines. All MIC values are expressed in mg/L [25].

^b^*L. Lactis* strains were classified as Resistant (R) if the MIC was higher than the EFSA cut-off values, and as Susceptible (S) if the MIC was lower than the cut-off values.

**Table 3:** Amino acid profile of MRS broth and CFS of H10 and M9.

Amino acids	MRS broth	SNU-H10 CFS	SNU-M9 CFS
Aspartic acid	87.56 ± 1.56	115.62 ± 1.43^a^	115.97 ± 0.60^a^
Serine	156.96 ± 2.89	182.67 ± 3.28^a^	179.83 ± 1.74^a^
Glutamine	7.15 ± 0.25	88.36 ± 1.34^a^	95.39 ± 0.63^a^
Histidine	41.37 ± 0.45	46.33 ± 0.51^a^	46.22 ± 0.21^a^
Glycine	534.92 ± 8.15	713.73 ± 15.78^a^	700.30 ± 9.89^a^
Threonine	143.04 ± 3.72	166.71 ± 1.23^a^	165.19 ± 1.13^a^
Citrulline	8.06 ± 0.14	63.44 ± 1.24^a^	68.15 ± 0.46^a^
Alanine	376.08 ± 7.44	526.24 ± 8.07^a^	514.24 ± 3.31^a^
GABA	21.59 ± 0.87	207.85 ± 3.83^a^	214.00 ± 2.71^a^
Tyrosine	43.65 ± 0.67	52.93 ± 1.04^a^	52.58 ± 2.02^a^
Valine	132.37 ± 2.57	188.43 ± 2.37^a^	186.44 ± 0.79^a^
Methionine	51.28 ± 0.77	60.07 ± 0.86^a^	59.37 ± 0.37^a^
Tryptophan	59.27 ± 1.39	67.51 ± 0.76^a^	66.47 ± 0.15^a^
Phenylalanine	158.97 ± 4.72	186.26 ± 3.22^a^	185.32 ± 1.06^a^
Isoleucine	115.29 ± 3.18	138.80 ± 2.17^a^	137.74 ± 0.46^a^
Ornithine	23.16 ± 0.84	316.16 ± 5.36^a^	318.70 ± 1.92^a^
Leucine	305.91 ± 6.98	340.25 ± 4.92^a^	337.34 ± 1.87^a^
Proline	37.87 ± 1.65	42.25 ± 2.35^a^	43.90 ± 1.76^a^
Glutamic acid	388.82 ± 8.43	175.52 ± 3.89^a^	156.13 ± 0.66^a^
Asparagine	86.30 ± 1.53	64.02 ± 1.06^a^	61.97 ± 0.11^a^
Arginine	382.43 ± 8.91	n.a.^b^	n.a.^b^
Taurine	15.07 ± 0.75	14.80 ± 0.49	14.99 ± 0.15
Lysine	200.66 ± 5.96	206.05 ± 3.89	204.17 ± 1.53

Amino acid concentrations are presented as mean ± standard deviation (n =3).

^a^Statistically significant difference in amino acid levels compared to MRS broth (*p* < 0.05).

^b^n.a. Not available; amino acid not detected in the sample.

Abbreviations: MRS broth, De Man–Rogosa–Sharpe, Sharpe broth; CFS, cell-free supernatant; GABA, γ-aminobutyric acid

**Table 4:** List of probiotic marker genes related to acid and alkaline stress?, bile salt tolerance, adhesion, and antioxidant activity identified in the genomes of SNU-H10 and M9.